# Design of a multi-signature ensemble classifier predicting neuroblastoma patients' outcome

**DOI:** 10.1186/1471-2105-13-S4-S13

**Published:** 2012-03-28

**Authors:** Andrea Cornero, Massimo Acquaviva, Paolo Fardin, Rogier Versteeg, Alexander Schramm, Alessandra Eva, Maria Carla Bosco, Fabiola Blengio, Sara Barzaghi, Luigi Varesio

**Affiliations:** 1 Laboratory of Molecular Biology, G. Gaslini Institute, Genoa 16147, Italy; 2 Department of Human Genetics, Academic Medical Center, University of Amsterdam, Amsterdam 1100, The Netherlands; 3 Department of Pediatric Oncology and Hematology, University Children's Hospital Essen, Essen 45122, Germany

## Abstract

**Background:**

Neuroblastoma is the most common pediatric solid tumor of the sympathetic nervous system. Development of improved predictive tools for patients stratification is a crucial requirement for neuroblastoma therapy. Several studies utilized gene expression-based signatures to stratify neuroblastoma patients and demonstrated a clear advantage of adding genomic analysis to risk assessment. There is little overlapping among signatures and merging their prognostic potential would be advantageous. Here, we describe a new strategy to merge published neuroblastoma related gene signatures into a single, highly accurate, Multi-Signature Ensemble (MuSE)-classifier of neuroblastoma (NB) patients outcome.

**Methods:**

Gene expression profiles of 182 neuroblastoma tumors, subdivided into three independent datasets, were used in the various phases of development and validation of neuroblastoma NB-MuSE-classifier. Thirty three signatures were evaluated for patients' outcome prediction using 22 classification algorithms each and generating 726 classifiers and prediction results. The best-performing algorithm for each signature was selected, validated on an independent dataset and the 20 signatures performing with an accuracy > = 80% were retained.

**Results:**

We combined the 20 predictions associated to the corresponding signatures through the selection of the best performing algorithm into a single outcome predictor. The best performance was obtained by the Decision Table algorithm that produced the NB-MuSE-classifier characterized by an external validation accuracy of 94%. Kaplan-Meier curves and log-rank test demonstrated that patients with good and poor outcome prediction by the NB-MuSE-classifier have a significantly different survival (p < 0.0001). Survival curves constructed on subgroups of patients divided on the bases of known prognostic marker suggested an excellent stratification of localized and stage 4s tumors but more data are needed to prove this point.

**Conclusions:**

The NB-MuSE-classifier is based on an ensemble approach that merges twenty heterogeneous, neuroblastoma-related gene signatures to blend their discriminating power, rather than numeric values, into a single, highly accurate patients' outcome predictor. The novelty of our approach derives from the way to integrate the gene expression signatures, by optimally associating them with a single paradigm ultimately integrated into a single classifier. This model can be exported to other types of cancer and to diseases for which dedicated databases exist.

## Background

Neuroblastoma is the most common pediatric solid tumor, deriving from ganglionic lineage precursors of the sympathetic nervous system [1]. It is diagnosed during infancy and shows notable heterogeneity with regard to histology and clinical behavior, ranging from rapid progression associated with metastatic spread and poor clinical outcome to spontaneous, or therapy-induced regression into benign ganglioneuroma. Age at diagnosis, stage, histology, DNA index, chromosomal aberrations, and amplification of the N-myc proto-oncogene (*MYCN*) are clinical and molecular risk factors commonly combined to classify patients into high, intermediate and low risk subgroups on which current therapeutic strategy is based. About fifty percent of high risk patients die despite treatment making the exploration of new and more effective strategies for improving stratification mandatory [2].

The availability of genomic profiles improved our prognostic ability in many types of cancers including neuroblastoma [3]. Several groups have developed gene expression-based approaches to stratify neuroblastoma patients [4-10]. One approach for patients stratification is to apply feature selection techniques to the patients' datasets to derive gene expression signatures representative of either biological processes related to tumor progression (biology-driven), such as tumor hypoxia [11,12], risk estimation (risk-driven) [9] or unsupervised clustering. Several groups used gene expression-based approaches to stratify neuroblastoma patients. Prognostic gene signatures were described and neuroblastoma classifiers were trained to predict the risk class and/or patients 'outcome [4-10].

Prognostic gene expression signatures have often similar performances despite the lack of gene overlapping suggesting that they relate to a common biological feature but derive from a highly variable environment [13]. Combination of the information contained in these signatures should improve the accuracy and/or the predictive power suggesting the potential application of ensemble learning approaches to increase not only the accuracy of the classification, but also the confidence of the results. Ensemble methods were originally developed to enhance classification performance [14] and have been recently applied to biomarkers identification and feature selection [15]. The general idea of this family of techniques consists in combining lots of different models in a global, more robust, model. The task of combining existing neuroblastoma gene expression signatures is rather complex because they were designed by biology or risk-driven approaches, hence with different finalities and applicability. Furthermore, these signatures were derived using different platforms and datasets thus preventing a straightforward integration. The problem of merging signatures or datasets was recently addressed in breast cancer where it was shown that multiple signatures can lead to robust prognostic when combined to clinical variables and large databases of gene expression [16]. Furthermore, Nuyten et al. demonstrated the relevance of combining biological gene expression signatures into an independent predictor for outcome in breast cancer patients [17]. Recently, Fan et al. [18] reported the generation of a prognostic model combining hundreds of gene expression signatures to clinical-pathological factors utilizing the Least Absolute Shrinkage and Selection Operator method and a Cox proportional hazards approach.

These results raised the question as to whether we could design an ensemble-based learning approach suitable for integrating gene expression signatures of neuroblastomas tumors where patients stratification is critical for the choice of treatment. Each tumor type has unique biological an clinical attributes and the best performing approaches must be designed accordingly. The potential problem of merging previously established signatures is that their implementation takes them out of the context in which they were generated in terms of experimental platform, dataset, paradigm and finality. We addressed this issue by introducing a meticulous selection of the algorithms for optimal performance of each signature and by building the final single classifier on the predictions rather than on gene expression values.

## Results

### Datasets

**Figure 1:**
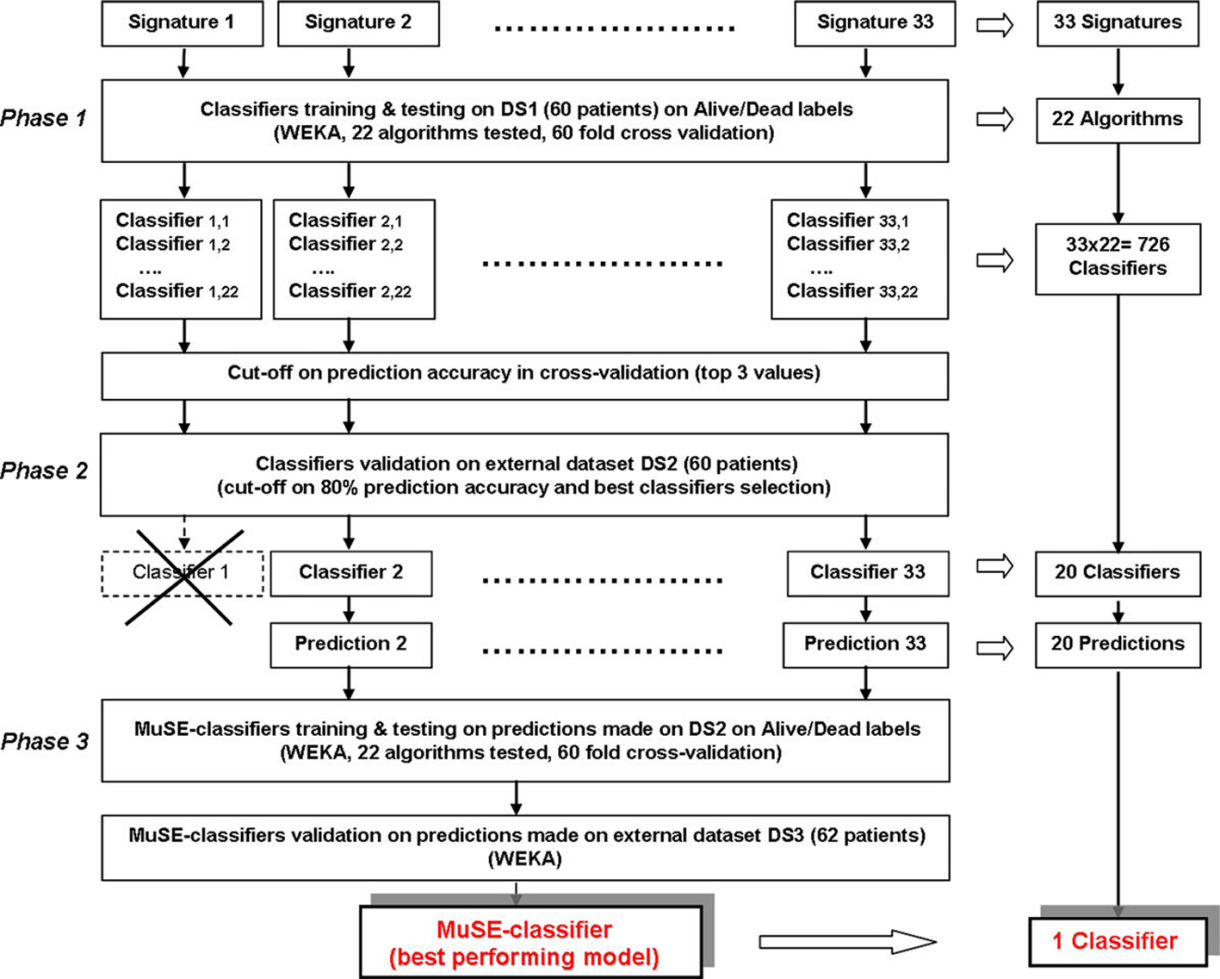
**NB-MuSE-classifier construction**. Workflow of the steps involved in the construction of the NB-MuSE classifier merging the information of 20 signatures matched to the optimal paradigm for outcome classification. The process can be subdivided into three main phases: 1) single signature classifiers generation, 2) classifiers filtering on performance features and, 3) Neuroblastoma Multi-Signature Ensemble classifier (NB-MuSE-classifier) training and validation. The dataset was subdivided into three different subsets: DS1(60 patients) to train the signatures, DS2 (60 patients) to externally validate single-signature classifiers and to train the NB-MuSE-classifier, and DS3(62 patients) to externally validate the NB-MuSE-classifier. The products of the procedure are indicated on the right side of the figure. 60 fold cross validation refers to leave one out cross-validation (LOOCV).

We aimed at integrating the prognostic information contained in different neuroblastoma gene signatures heterogeneous with respect to the origin, feature selection, platform and dataset. The method has been applied on a dataset containing the gene expression profiles of 182 neuroblastoma patients (detailed in the Material and Methods section) with a distribution of the common risk factors (Table 1) compatible with what reported in the literature [19]. The process can be subdivided into three main phases (Figure 1): i) single signature classifiers generation, ii) classifiers filtering on performance figures and, iii) NB-MuSE-classifier training and validation. The patients' cohort was subdivided into three independent datasets (DS): DS1 (60 patients) to train the signatures, DS2 (60 patients) to externally validate single-signature classifiers and to train the NB-MuSE-classifier, and DS3 (62 patients) to externally validate the NB-MuSE-classifier. The characteristics of these datasets are detailed in the additional file 9. The signatures considered in this work were selected from the literature by Medline search of articles published after year 2002. We selected, by visual screening, the articles effectively describing neuroblastoma gene signatures. This process led to the identification of 39 neuroblastoma-related gene signatures, either risk-or biology-driven, of which 33 reported a detailed gene list and were used for the analysis [1,4-10,12,20-43].

**Table 1:** Clinical characteristics of the 182 neuroblastoma patients analyzed

**Risk groups**^**a**^		**Patients**^**b**^	**Distribution (%)**^**c**^
**INSS stage**	1	42	23,1
	2	24	13,2
	3	23	12,6
	4	70	37,9
	4s	23	13,2

**Age at diagnosis**	< = 1 year	92	51,1
	> 1 year	90	48,9

**MYCN status**	Normal	153	84,1
	Amplified	29	15,9

**5 years survival**	Dead	55	29,7
	Alive	127	70,3

^a ^Common risk groups and categories stratifying neuroblastoma patients.

^b ^The total number of patients in each group was 182. The number of patients in each subdivision is shown.

^c ^Percentage relative to the total number of patients in each risk group.

### Phase 1. Single signature classifier generation

Next, we evaluated the ability of each selected signature to predict patients outcome (Figure 1). This step, although labor intense, is critical to assess performance of each of signatures and to filter out those poorly informative. Each of the 33 signatures was used to train machine learning classifiers predicting neuroblastoma patients' outcome. A panel of 22 classification paradigms implemented by the WEKA package (for ref see [44]) was tested for each signature to select the best possible classifier. For each signature, the expression data of the 60 patients of dataset DS1 and the associated labels ("Alive"/"Dead") were used to train a classifier in a leave-one-out cross-validation (LOOCV) framework. Thus, 726 classifiers, were generated combining 22 paradigms and 33 signatures (Figure 1).

### Phase 2. Classifiers filtering on performance figures

The following step consisted in the external validation of every classifier for each signature by the application of the models to the independent group of 60 patients included in the DS2. Only the signatures performing with an accuracy > = 80% were retained and the others excluded from the analysis to avoid background noise. A literature search indicated that 80% accuracy was the lower limit considered relevant for patients' classification. This process filtered-out 13 signatures. Details on the 20 gene signatures that were retained an on the gene-associated probesets are shown in the additional file 7. There was little overlapping in gene representation among the 20 signatures composed of a total of 741 different genes of which only 128 were present in 2 or more signatures and none was common to all of them. The most frequent gene was *NTRK1 *(7 signatures) followed by *MAPT *(6 signatures), *MYCN*, *TYMS*, *VEGFA *(5 signatures). These signatures differ for feature selection criteria as shown by the biology-driven (referred to as "biology") or risk-driven (referred to as "stratification") categories listed in Table 2. Despite the differences among these two groups, we have almost the same number of signatures selected for each category (Table 2). These 20 gene signatures were matched individually with the corresponding best performing classifier and included in the third phase of the analysis. Classification performance for the 20 classifiers on the independent DS2 validation set ranged from 80% to 87% (Table 3). Twelve out of 22 paradigms were utilized with the Multi Layer Perceptron being the most represented but there was no obvious bias towards a defined category of algorithms. The number of genes per signature is variable (range 9-120, additional file 7) but it does not seem influential on the predicting power. These results generated the prediction matrix of the selected signatures and related algorithms (Additional file 2). In summary, the 20 signatures shown in Table 2 are, individually, the most informative for patients' outcome prediction in our framework and their associated classifier was used to generate the NB-MuSE-classifier.

**Table 2:** Twenty signatures selected for NB-MuSE-classifier construction

Signature*	PMID	Reference	Genes^	Category^^	Features°
Chen	19921788	[22]	50	biology	MYCN
De Preter II	16989664	[1]	73	biology	Neuroblast transformation
Di Pietro	19402918	[23]	33	biology	Apoptosis
Fardin	20624283	[12]	62	biology	Tumor hypoxia
Fransson	17044048	[24]	10	biology	Chromosome 1p36
Fredlund	18780787	[25]	103	biology	MYCN expression
Hahn	18607002	[26]	14	biology	Histone deacetylase
McArdle	15090470	[29]	10	biology	Chromosome 11q
Nevo II	15081541	[31]	13	biology	CXCR4 receptor
Oe	15992370	[28]	117	biology	response to NGF
Shimada	17941064	[27]	5	biology	Biomarkers
*Asgharzadeh*	*16954472*	[20]	*44*	*stratification*	*MYCN non amplified*
*Benard*	*19383347*	[21]	*37*	*stratification*	*age groups*
*De Preter I*	*20179214*	[10]	*42*	*stratification*	*Risk/outcome*
*Fischer*	*16951229*	[8]	*17*	*stratification*	*St4 vs St4s*
*Nevo II*	*19739072*	[30]	*95*	*stratification*	*St1 vs St4*
*Oberthuer*	*17075126*	[7]	*108*	*stratification*	*Risk/outcome*
*Ohira*	*15837623*	[6]	*39*	*stratification*	*Risk/outcome*
*Vermeulen*	*19515614*	[9]	*59*	*stratification*	*Risk/outcome*
*Wei*	*15466177*	[4]	*12*	*stratification*	*Risk/outcome*

* First author's name of the paper describing the neuroblastoma-related signature used. The signatures predicate patients' outcome with an accuracy > 80%.

^^ ^Number of genes comprising the signature.

^^^ ^Categories indicative of the natureof the signature. Biology: the signature discriminates biologically defined situations. Stratification: the signature discriminates classes of tumors derived from patients' stratification.

° Features: Indications on the nature of the biological questions asked or stratification criteria used. St = INSS stage.

**Table 3:** Merging individual classifiers into the NB-MuSE classifier

Classifier*	External validation	Paradigm°	Function
			
	Accuracy (%)^		
Chen	85	BayesNet	Learns Bayesian nets
Di Pietro	83	BayesNet	Learns Bayesian nets
Fredlund	80	ClassificationViaRegression	Class is binarized and one regression model is built for each class value
Asgharzadeh	83	ComplementNaiveBayes	Builds a complement Näive Bayes classifier
Fransson	85	ComplementNaiveBayes	Builds a complement Näive Bayes classifier
De Preter II	87	IBk	k-nearest-neighbors classifier
Wei	83	IBk	k-nearest-neighbors classifier
De Preter I	83	KStar	Nearest neighbor with generalized distance function
Oberthuer	87	Logistic	Builds linear logistic regression models
Hahn	82	MultiLayerPerceptron	Backpropagation neural network
McArdle	80	MultiLayerPerceptron	Backpropagation neural network
Oe	80	MultiLayerPerceptron	Backpropagation neural network
Nevo II	87	NaiveBayes	Standard probabilistic Näive Bayes classifier
Shimada	80	NBTree	Builds a deciosion tree with Näive Bayes classifier at the leaves
Vermeulen	85	NBTree	Builds a decision tree with Näive Bayes classifier at the leaves
Ohira	85	RandomForest	Constructs random forest
Fischer	81	SimpleLogistic	Builds linear logistic regression models with built-in attribute selection
Fardin	83	Voted Perceptron	Voted perceptron algorihtm
Nevo I	80	Voted Perceptron	Voted perceptron algorihtm

**NB-MuSE**	**94**	DecisonTable	Builds a simple decision table majority classifier

* Classifier associated to the signature described in the paper whose first name is listed.

^^ ^External validation on the DS3 dataset.

° Paradigms that gave the top performance in external validation.

### Phase 3. Neuroblastoma Multi-Signature Ensemble classifier training and validation

In the third phase of our analysis we combined the 20 predictions associated with the corresponding signature into a single outcome prediction. For this purpose, we trained a new classifier (NB-MuSE-classifier) on the previously generated dataset containing the 20 prediction applied to the DS2 patients. Similarly to the training and validation step, 22 algorithms were tested to sort out the best performing (Additional file 8). The performance of the NB-MuSE-classifier was validated on the independent dataset DS3. The classification accuracy of the classifiers that were used in the process leading to the selection of the best performing algorithm is detailed in the Additional file 8. The best performance was obtained by the Decision Table algorithm that produced a classifier characterized by an external validation accuracy of 94% (Table 3 and Additional file 8). This accuracy is greater than that shown by the individual algorithms within the context of the current framework and it represents an excellent predictor of neuroblastoma outcome based on gene expression profile. The predictions of the NB-MuSE-classifier relative to that of the individual signatures from which it was derived is shown in the Additional file 8. In conclusion, we combined the predictive power of different signatures to merge survival categories into a single classifier predicting, with high accuracy, the outcome of neuroblastoma patients.

### Evaluation of multi-step classification process

**Figure 2:**
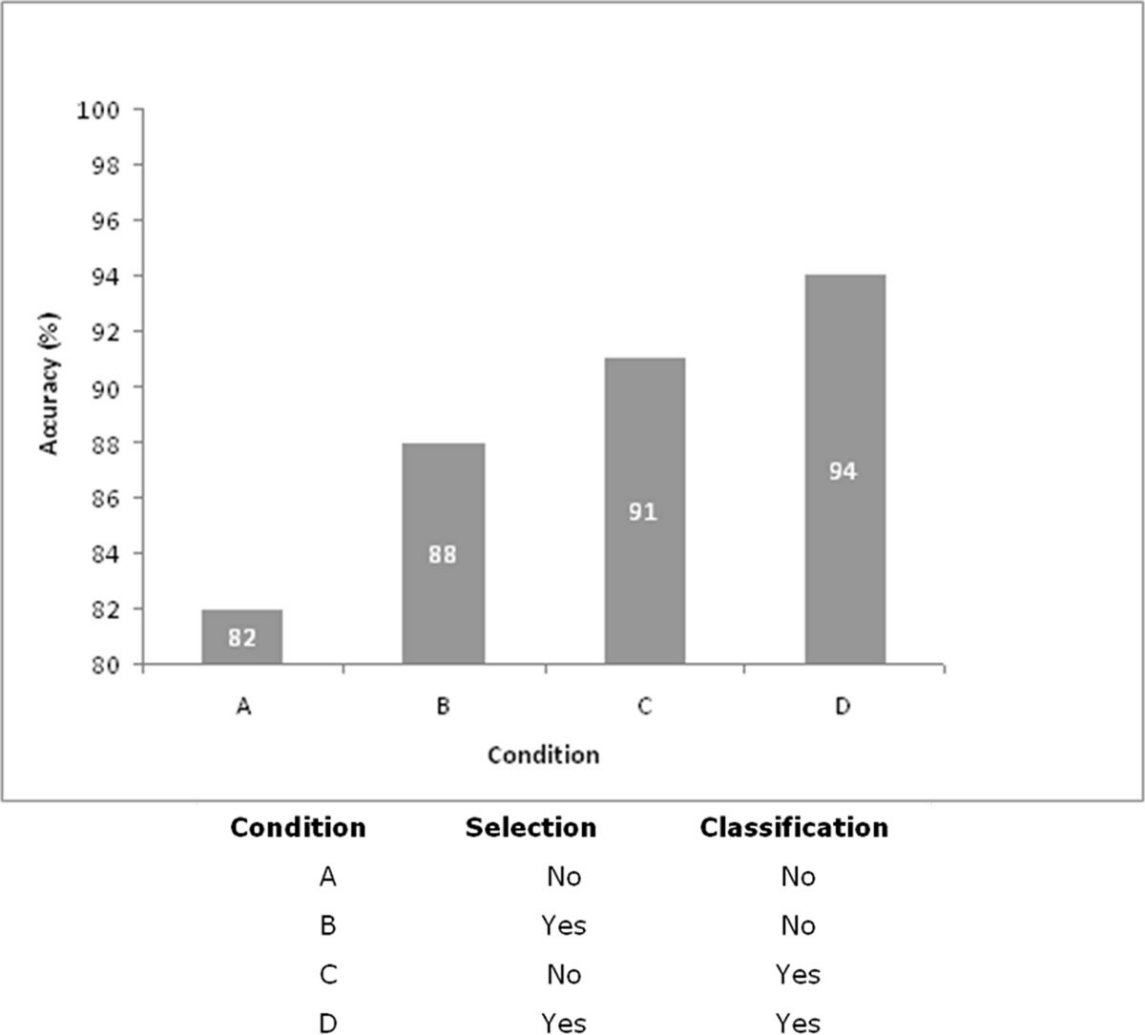
**Evaluation of the multistep classifier generation process**. The accuracy of NB-Muse classifier was measured when the selection and/or classification steps were omitted from the procedure. The four resulting possible conditions are indicated with letters from A to C as detailed in the figure. The percent accuracy for each condition is shown in the corresponding bar.

Our method included a filtering step dedicated to the exclusion of signatures characterized by an associated low performing classifier accuracy (< 80%) to remove prediction results characterized by a high classification error and/or to reduce data noise. The second classification step was introduced to identify an optimal labeling method. Similar conclusions could be achieved by evaluating each of the two labels frequencies in the prediction results of the single-signature classifiers. In order to assess the need/validity of these steps, we re-applied our method switching off the filtering step and substituting the second classification step with label frequencies evaluation. As shown in Figure 2, we obtain optimal performance figures by the inclusion of the filtering step and the use of the second classification step. In fact, when both filtering and classification were switched off, the procedure achieves an accuracy of 82%, lower than most e single-signature classifiers (Table 3). When filtering is preserved and classification is turned off, we obtained an accuracy of 88%. Finally, by turning off the filtering alone, we achieve an accuracy of 91% still lower that that obtained by the completed method. These results demonstrate that the filtering on performances of single-signature classifiers helps to limit classification error. Furthermore, the application of supervised classification approaches is required to obtain an optimal interpretation of highly complex biological data.

### Clinical impact of the results

**Figure 3:**
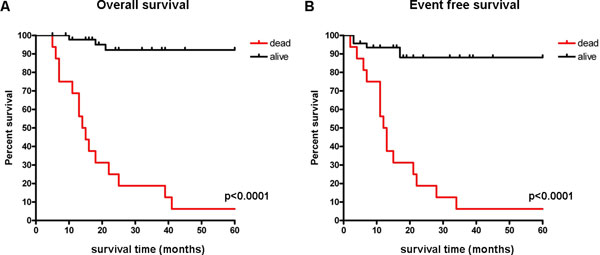
**Kaplan-Meier and log-rank analysis of patients stratified according to the NB-MuSE classifier**. Kaplan-Meier and log-rank analysis for the 62 neuroblastoma patients belonging to the external validation dataset. 5-years overall survival (A) and event free survival (B) of patients stratified according to the NB-MuSE classifier. Red and black curves represent poor and good outcome patients respectively. The p-value of the log-rank test is shown.

The overall- and event free-survival of the patients divided according to the NB-MuSE-classifier are shown in Figure 3. Kaplan-Meier curves and log-rank test demonstrated that patients with good and poor outcome prediction have a significantly different survival (p < 0.0001). Interestingly, the 4 mislabeled patients corresponding to the 6% error of NB-MuSE-classifier on DS3 are characterized by INSS stage 4 tumors whereas 100%accuracy was reached in classifying the outcome of stages 1,2,3 and 4s tumors (data not shown). In agreement with these results, the Kaplan Mayer curves and log-rank test of patients with stages 1,2,3 and 4s tumors showed a very good separation of outcome (Additional file 4). In contrast, MuSE signature was much less effective in stratifying st4 patients (Additional file 3). Analysis of patients positive for *MYCN *rearrangement failed to show a significant stratification by MuSE classifier (Additional file 5) as expected by the small sample size. In contrast, There was a good separation of outcome in patients with *MYCN *non amplified tumors (Additional file 6) although not as significant as that of the whole cohort. We conclude that NB-MuSE-classifier is very effective in stratifying neuroblastoma patients outcome. We feel that combination of NB-MuSE-classifier with INSS staging may be particularly accurate in predicting the progression of the disease. However, more data should are needed to prove this point.

## Discussion

We designed a new prognostic model based on a neuroblastoma classifier, NB-MuSE, that predicts patients' outcome by merging the biological and prognostic information of published gene expression signatures, assessed by a panel of machine learning algorithms, into a single outcome predictor. We examined every neuroblastoma-related signature described in the literature since 2002 without consideration for the purpose for which it was generated or the gene expression platform used. We took the blind screening approach to avoid biases and to include biology-driven signatures, not previously tested for patients stratification, in addition to risk-based signatures. We identified 33 signatures, complete of gene lists, suitable for our study. Patients' outcome was the final readout of the classifier and we had to develop a strategy to filter out poorly information signatures contributing to the background noise. We developed a multi-algorithm screening and an 80% accuracy filter for signature selection. This essential step was based on the overproduce-and-select approach in which a pool of classifiers are spawned and then optimally selected on-the-fly by monitoring accuracy of prediction on an external dataset. We evaluated 22 machine learning algorithms for outcome prediction on the 33 signatures generating 726 prediction to be evaluated for accuracy on an independent dataset. We selected the signatures for which we identified at least one algorithm performing with an accuracy > 80%. Exclusion of a signature from this analysis indicated that we did not identified an algorithm capable of translating those signatures into a predictor in our cohorts or that the signatures were not related to patients' outcome but it does impact on the relevance of those genes in the contest of the original publication. Eleven out of thirty three signatures were discarded. We then matched each of the remaining 20 signatures with the best performing algorithm among those with > 80% accuracy to generate signature specific outcome prediction classifier. In essence, we transformed 20 datasets each with 60 instances (patients) and numeric attributes (probesets expression value) into one dataset with 60 instances and 20 nominal "alive" or "dead" attributes (one per selected signature). The latter dataset could then be used as input to train the new NB-MuSE-classifier merging all the signature information. 22 algorithms were tested to select the best performing which was the Decision Table which builds a simple decision table majority classifier and evaluates features subsets using best-first search and can use cross validation for evaluation (for review see [44]). Performance can be evaluated by many parameters and there is heterogeneity in the performance of the various algorithms tested as shown by the Additional file 8. The Decision Table algorithm was chosen because it showed maximal accuracy, but other parameters could have been selected to highlight other features like sensitivity or specificity. Ensemble learning approaches have proven to exceed average classifier performance [45]. Our strategy utilizes such strategy to produce a flexible tool merging gene expression signatures overcoming the limitations imposed by specific environments in which they were generated. We observed that, in the absence of signature/algorithm filtering, the accuracy of our classifier fell below 82% a level that was lower than that achieved by individual classifiers. The importance of including these steps in model generation procedures to obtain a more robust and better performing classifier was recently reported [46]. Optimization and filtering is quite labor intense and was not considered, for example, in breast cancer studies merging hundred of gene expression signatures to build classifiers [18]. The high number of signatures available in breast cancer may balance the avoidance of filtering out poorly informative signatures. An automated implementation of this process can be envisioned if this approach was exported to larger lists of signatures.

The accuracy of NB-MuSE-classifier on external validation was 94%, a value that is very high from the biologic stand point. Although there is no logical reason why it cannot be higher, it is difficult to envision a much better precision from a biological standpoint considering the variability of the experimental and clinical data. On the other hand, there is no limit to the number of signatures that can be derived with biological questions in mind. Our model offers a reliable way to keep merging this information into an outcome classifier that will be more robust even if not much more accurate. It is noteworthy that the misclassified patients are grouped in the stage 4 category in agreement with the fact that prognosis of this stage is traditionally difficult. We can speculate that combination of the information of stage and NB-MuSE-classifier could be particularly effective in predicting outcome in patients with localized tumors (stage 1-3) or stage 4s Survival analysis if this group of patients supports this claim showing excellent outcome separation superior to that observed on the whole cohort. however, more patients will have to be tested to substantiate this claim. Similar analysis performed on patients with *MYCN *amplified tumors showed a significant outcome stratification although not as good as that observed with the whole cohort. We are working on strategies for comparing neuroblastoma gene expression dataset obtained with different platforms in order build a larger data set to address question on smaller groups of patients. We are among the few focusing on the question of merging heterogeneous gene expression signatures to predict outcome. To limit the variability, we considered only gene expression data generated by microarray analysis of the primary neuroblastomas using the Affymetrix platform U133plus2 and we put together 182 primary neuroblastomas, a cohort that is large for this kind of tumor. On the other hand, there was no restriction on the technology used to generate the signatures that turned out to be quite heterogeneous demonstrating that our multistep approach a is suited to work across experimental platforms. This aspect is very important particularly in the field of rare tumors, such as pediatric tumors, where it is extremely difficult to build large homogeneous gene expression datasets and where we may envision that the developing signatures will be based on new experimental platforms.

Affymetrix platform differs largely from the those used in the studies reporting the single classifiers (e.g. two-color gene-expression data from different technological platforms, QPCR analyses etc.). In addition, some of the machine learning algorithms used in the original reports of the classifiers were not part of the panel used in the present study. This may explain discrepancies between the performances of individual signatures that were previously published and that calculated in this work. The problem of downplaying the performance of some signatures is partially offset by the discovery of the prognostic ability of other signatures, a feature not previously shown in the original publications. However, the possible advantage of the MuSE-classifier over presently existing classifiers cannot be easily quantified because we took individual signatures out of their original contest. Table 3 shows that merging signatures into a single classifier results in a predictor with very high accuracy but it does not imply that this value is maximal and considerations on the relative performance of MuSE versus other signatures is valid only in the contest of this work.

The discovery of outcome prediction ability of biology-driven signatures, never tested before for patients stratification, is a spinoff of the process of NB-MuSE-classifier generation. This was true for most of the biology-driven signatures comprising about half of those in the NB-MuSE-classifier [1,22-24,26-29,31] with the exception of those addressing the prognostic significance of hypoxia [12] and MYC pathway [25] that had already been validated in patients stratification. Our data bear direct evidence to the suggestion that the biology driven features, measured by the gene expression signatures, such as neuroblast transformation, apoptosis histone deacetylase etc. (Table 2) are strongly interconnected with the progression of the human disease and support the need for further research in this direction [47].

## Conclusions

We describe the design, generation and properties of the NB-MuSE-classifier based on an ensemble approach that merges heterogeneous, neuroblastoma-related gene signatures to blend their discriminating power, rather than their numeric values, into a single, highly accurate, patients' outcome predictor. The key of our method is merging several datasets with numeric attributes into one dataset with nominal "alive" or "dead" attributes. The latter dataset could then be used as input to train the new single classifier merging all of the prognostic information of individual signatures through a process which combines individual models into an ensemble of learned models. Inevitably, the framework leading to the MuSE-classifier implied taking the signatures out of the original contest and matching, for example, the genes with the Affymetrix platform probsets. Therefore, the performances calculated by us may be different from that originally reported and considerations on the relative performance should be limited to our framework. On the other hand, our approach showed that signatures can be successfully taken out of their contest retaining their prognostic value. Moreover, the process of NB-MuSE-classifier generation lead to the discovery of the effectiveness of several biology-driven published, signatures to predict outcome suggesting that the biological features measured by such signatures could be mechanistically related to the progression of the human disease.

The novelty of our approach derives from the way to integrate the gene expression signatures, by optimally associating them with a single paradigm ultimately integrated into a single classifier. This approach was developed on a Neuroblastoma dataset. However, this model can be exported to other cancer types and to other diseases for which dedicated databases exist.

## Methods

### Patients

A total of 182 neuroblastoma patients belonging to four independent cohorts were enrolled on the bases of the availability of gene expression profile by Affymetrix GeneChip HG-U133plus2.0 and clinical and molecular information. Eighty-eight patients were collected by the Academic Medical Center (AMC; Amsterdam, Netherlands) [12]; 21 patients were collected by the University Children's Hospital, Essen, Germany and were treated according to the German Neuroblastoma trials, either NB97 or NB2004; 51 patients were collected at Hiroshima University Hospital or affiliated hospitals and were treated according to the Japanese neuroblastoma protocols [48]; 22 patients were collected at Gaslini Institute(Genoa, Italy) and were treated according to Italian AIEOP or European SIOPEN protocols. We utilized the gene expression profiles and associated clinical parameters available at the R2 repository [49] (AMC and Essen patients), at the BIT-neuroblastoma Biobank of the Gaslini Institute [50] of which Dr. Varesio coordinates the tumor molecular classification (Genova patients). The instigators who deposited the data in the R2 repository agree to use the data for this work. In addition, we utilized the data present on the public database at the Gene Expression Omnibus number GSE16237) for Hiroshima patients [48]. Informed consent was obtained in accordance with institutional policies in use in each country. In every dataset, median follow-up was longer than 5 years and tumor stage was defined according to the International Neuroblastoma Staging System. The clinical characteristics of the 182 neuroblastoma tumors are listed in Table 1. Good and poor outcome were defined as patient's status (alive or dead) 5 years after diagnosis. The 182 patients dataset was randomized and divided into three subsets (DS1, DS2, and DS3) consisting of 60, 60, and 62 patients respectively. The characteristics of the composition of these datasets are detailed in the Additional file 9. DS1 has been used to train the signatures, DS2 to externally validate the single-signature classifiers and to train the NB-MuSE-classifier, and DS3 for external validation of the NB-MuSE-classifier.

### Gene expression analysis

Gene expression profiles for the 182 tumors were obtained by microarray experiment using Affymetrix GeneChip HG-U133plus2.0 and the data were processed by MAS5.0 software according Affymetrix's guideline. For Gaslini's patients specimens total RNA was extracted using Trizol (Invitrogen Life technologies, Irvine, CA) according to the manufacturer's instructions. RNA was resuspended in diethyl pyrocarbonate-treated H_2_O (DEPC water), the physical quality control of RNA integrity was carried out by electrophoresis using Agilent Bioanalyzer 2100 (Agilent Technologies Waldbronn, Germany) and quantified by NanoDrop (NanoDrop Technologies Wilmington, Delawere USA). Total RNA was reverse transcribed into cDNA and biotin labeled according to the Affymetrix instructions (Affymetrix, SantaClara, CA). Fragmented cRNA was used for hybridization to Affymetrix HG-U133 Plus 2.0 arrays. Expression values were quantified, and array quality control was performed using the statistical algorithms implemented in Affymetrix Microarray Suite 5.0. The scale factors (SF) for all the hybridizations were within 1 SD of the mean (SF 1-3). To asses RNA integrity quality control and RNA digestion plot were used as implemented in the R package "Affy". Data of all datasets were processed by MAS5.0 software according Affymetrix's guideline.

### Neuroblastoma gene signatures selection

The Neuroblastoma related signatures were obtained by searching the relevant literature. Specifically, the papers were selected from the literature by Medline search using "neuroblastoma signatures" and "neuroblastoma expression profile" as keywords and limiting the search to articles published after year 2002. The process of selecting out the signatures is part of the workflow and it will be detailed in the results' section. As a result, 33 gene signatures were selected for NB-MuSE-classifier development [1,4-10,12,20-43]. To handle signatures not based on Affymetrix platform or lacking probesets values information, we associated, one Affymetrix GeneChip HG-U133plus2.0 probesets value to each gene of the signature by unpaired t-test on the entire dataset. The best probesets discriminating between the "alive" and "dead" class was picked to represent the unique gene names (Additional file 7). This selection criterion was preferred to other methods, such as mean, median or highest value, which can cause loss of information at the single probesets level and the relevance of the specific signature.

### NB-MuSE-classifier design

The NB-MuSE-classifier framework, is summarized in Figure 1. The WEKA package [44] has been chosen to perform all the training and validation steps in our analysis. Gene expression data have been used in linear scale in all the computations. In the first phase, each of the selected 33 gene signatures was used to generate a classifier trained to predict the neuroblastoma patients 'outcome. For each signature, the expression data of the 60 patients of dataset DS1 and the associated 60 "true" outcome labels ("Alive"/"Dead") was provided to WEKA to train a classifier in a leave-one-out cross-validation (LOOCV) framework. A panel of 22 classification algorithms available in Weka [44] was tested for each signature. The best performing algorithm was selected for each signature according to the prediction accuracy score obtained during LOOCV. As a cut-off, we chose the top three accuracy values for each gene signature. The next step consisted in the external validation of the selected classifiers for each signature by the application of the models to the patients gene expression values included in DS2. The best-performing model for each signature was selected. If the best-performing model had a prediction accuracy < 80%, the associated signature was discarded and no longer considered. The decision was made considering that relevant published classifiers have an accuracy that is generally > 80%. Some algorithms (Bagging, BayesLogisticRegression, ClassificationViaCluster, DecisionTable, FT, IB1, J48, LWL, RandomTree, ZeroR) did did not perform with sufficient accuracy in any of the signatures. The remaining 12 were utilized in the study (BayesNet, ClassificationViaRegression, ComplementNaiveBayes, IBk, KStar, Logistic, MultiLayerPerceptron, NaiveBayes, NBTree, RandomForest, SimpleLogistic, Voted Perceptron). Finally, we trained, tested, and validated the new MuSE-classifier that, during the training phase, takes into account the prediction produced by the application of the selected models to DS2 to produce a single prediction for each patient tested. A training dataset consisting of 60 patients has been assembled from the predictions generated by the 20 classifiers obtained in the previous phase on DS2 (Additional file 1) and, likewise, a validation dataset of 62 patients was assembled from the predictions performed by the same classifiers on DS3 (Additional file 2). Similarly to the training and validation steps performed for each gene signature during the first phase, 22 were tested to select optimal performance. The resulting best performing classifier is our NB-MuSE-classifier.

### Statistical analysis

The probability of overall survival (OS) and event-free survival (EFS) was calculated using Kaplan-Meier method, and the significance of the difference between Kaplan-Meier curves was calculated by the log-rank test using Prism 4.03 (GraphPad Software, Inc.). Accuracy, specificity, and sensitivity were computed to estimate the performance of the predictions performed in the various steps of the study.

## List of abbreviations

NB: Neuroblastoma; MuSE: Multi-Signature Ensemble; St: Stage; OS: overall survival; EFS: event free survival; DS: data set; LOOCV: leave-one-out cross-validation.

## Competing interests

The authors declare that they have no competing interests.

## Authors' contributions

AC, MA conceived of the study, participated in its design, helped to draft the manuscript. AE, MCB, SB, RV, AS participated in its coordination. FB carried out the microarray data analysis. PF conceived of the study, performed the statistical analysis and helped to draft the manuscript. LV supervised the study and wrote the manuscript.

## Supplementary Material

Additional file 1 **External validation of single signature classifiers**. Predictions performed on DS2, (60 patients) dataset by the 20 signatures associated to the best individual classifiers selected during the first phase are assembled in a prediction matrix and shown together with the true outcome. A: alive, D: dead. The signatures are indicated by the initials of the first author of the manuscript. The associated references are as follows: DIP: Di Pietro [23], FI: Fischer [8], FR: Fredlund [25], NE I: Nevo [30], OB: Oberhuer [7], OH: Ohira [6], WE: Wei [4], DEP I: De Preter [10], FA: Fardin [12], OE: Oe [28], SH: Shimada [27], HA: Hahn [26], CH: Chen [22], DEP II: De Preter II [1], BE: Benard [21], FR: Fransson [24], MCA: McArdle [29], AS: Asgharzadeh [20], NE II: Nevo II [31], VE: Vermulen [9]. Click here for file

Additional file 2 **NB-MuSE-classifier external validation**. Predictions performed on DS3, 62 patients dataset. The best individual-signature classifiers selected during the first phase have been assembled in a prediction matrix used for the external validation of the NB-MuSE-classifier. The "Actual" column represent the true Alive/Dead label for each patient. The "MuSE" column represent the outcome of the NB-MuSE-classifier in external validation. A: alive, D: dead. The signatures are indicated by the initials of the first author of the manuscript (see legend to additional file 1). Click here for file

Additional file 3 **Kaplan-Meier and log-rank analysis of patients with Stage 4 tumors stratified according to the NB-MuSE classifier**. Kaplan-Meier and log-rank analysis for INSS Stage 4 neuroblastoma patients belonging to the external validation dataset. 5-years overall survival (left) and event free survival (right) of patients stratified according to the NB-MuSE classifier. Red and black curves represent poor and good outcome patients respectively. The p-value of the log-rank test is shown. Click here for file

Additional file 4 **Kaplan-Meier and log-rank analysis of patients with localized and Stage 4s tumors stratified according to the NB-MuSE classifier**. Kaplan-Meier and log-rank analysis for INSS Stage 1,2,3 and 4s neuroblastoma patients belonging to the external validation dataset. 5-years overall survival (left) and event free survival (right) of patients stratified according to the NB-MuSE classifier. Red and black curves represent poor and good outcome patients respectively. The p-value of the log-rank test is shown. Click here for file

Additional file 5 **Kaplan-Meier and log-rank analysis of patients with *MYCN *amplified tumors stratified according to the NB-MuSE classifier**. Kaplan-Meier and log-rank analysis for neuroblastoma patients with *MYCN *amplified tumors belonging to the external validation dataset. 5-years overall survival (left) and event free survival (right) of patients stratified according to the NB-MuSE classifier. Red and black curves represent poor and good outcome patients respectively. The p-value of the log-rank test is shown. Click here for file

Additional file 6 **Kaplan-Meier and log-rank analysis of patients without *MYCN *amplification in the tumors stratified according to the NB-MuSE classifier**. Kaplan-Meier and log-rank analysis for neuroblastoma patients with *MYCN *not amplified tumors belonging to the external validation dataset. 5-years overall survival (left) and event free survival (right) of patients stratified according to the NB-MuSE classifier. Red and black curves represent poor and good outcome patients respectively. The p-value of the log-rank test is shown. Click here for file

Additional file 7 **Probesets and genes composition of the 20 signatures selected for building the MuSE classifier**. 20 signatures, out of 33 tested, were selected representing those performing with an accuracy > = 80. The gene composition (Gene Name) and matching probeset (Affymetrix) are shown. The choice of the probesets was made according to the criteria described in the Material and Methods' section. The signatures are indicated by the initials of the first author of the manuscript (see legend to additional file 1). Click here for file

Additional file 8 **Best performing algorithms tested to build the MuSE classifier**. The classification accuracy of the models that were used in the process leading to the selection of the best performing algorithm are shown together with the parameters describing the performances of each. The 18 algorithms that gave a meaningful separation are reported. ClassificationViaClustering, FT, BayesLogisticRegression and MBtree did not give a significant separation and are not shown. The algorithms are described and implemented in WEKA package [44]. The signatures are indicated by the initials of the first author of the manuscript (see legend to additional file 1). Click here for file

Additional file 9 **Characteristics of the datasets**. The 182 patients dataset was divided randomly into three subsets (DS1, DS2, and DS3) consisting of 60, 60, and 62 patients respectively. The characteristics of the composition of these datasets are shown. For each DS, the composition in terms of INSS stage representation, MYCN amplification, source of the data (AMC: Amsterdam; ESS: Essen; HI: Hiroshima; IGG: Genoa, for details see Material and Methods), age at diagnosis and Overall survival. Click here for file
