# Supplementary figures and images for: Design of a multi-signature ensemble classifier predicting neuroblastoma patients' outcome
Source: BMC Bioinformatics. 2012 Mar 28;13(Suppl 4):S13. doi: 10.1186/1471-2105-13-S4-S13 (PMC3314564; doi:10.1186/1471-2105-13-S4-S13)

## ST4

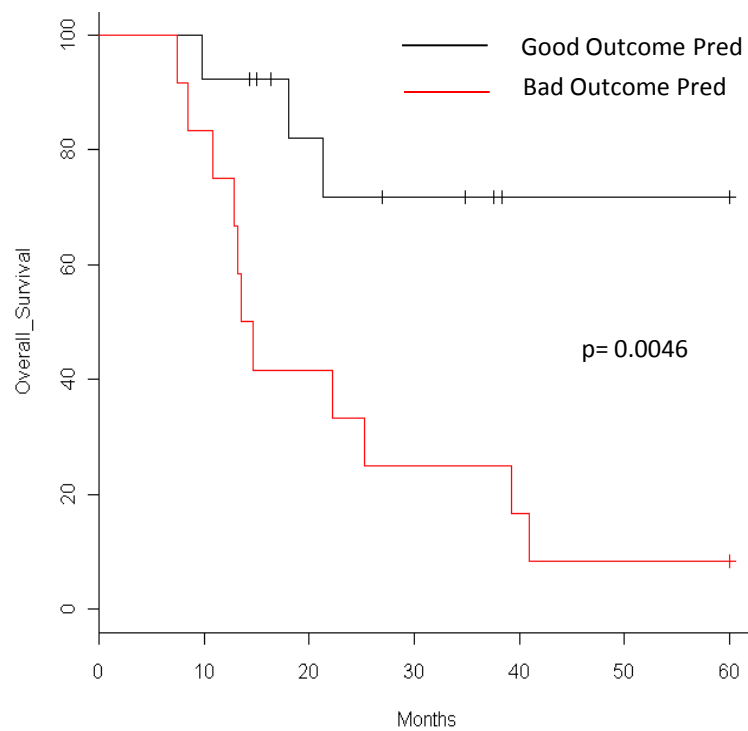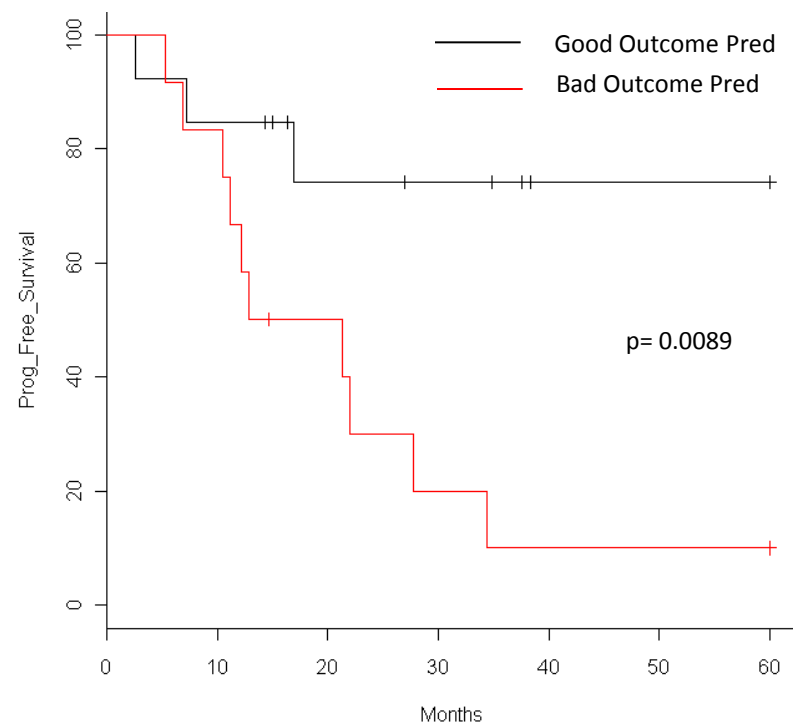

## ST4

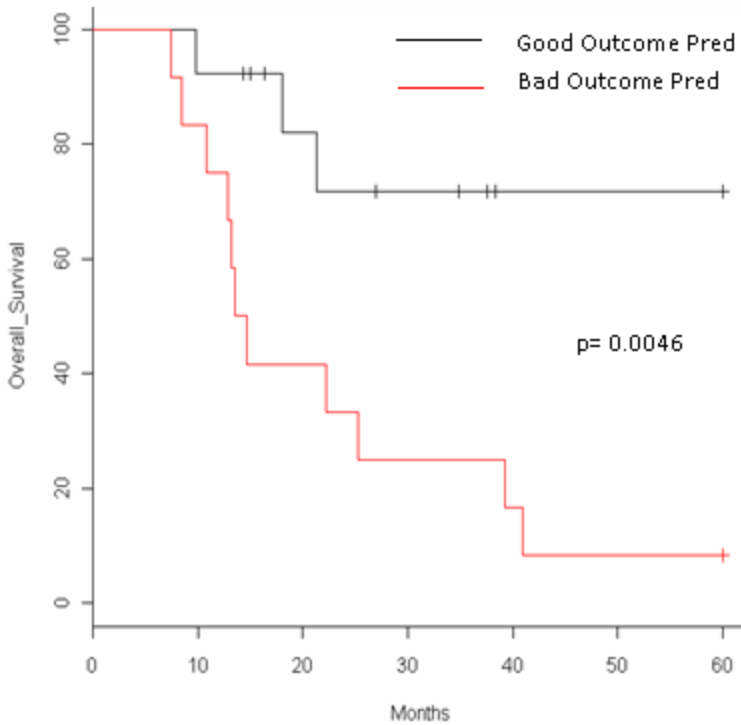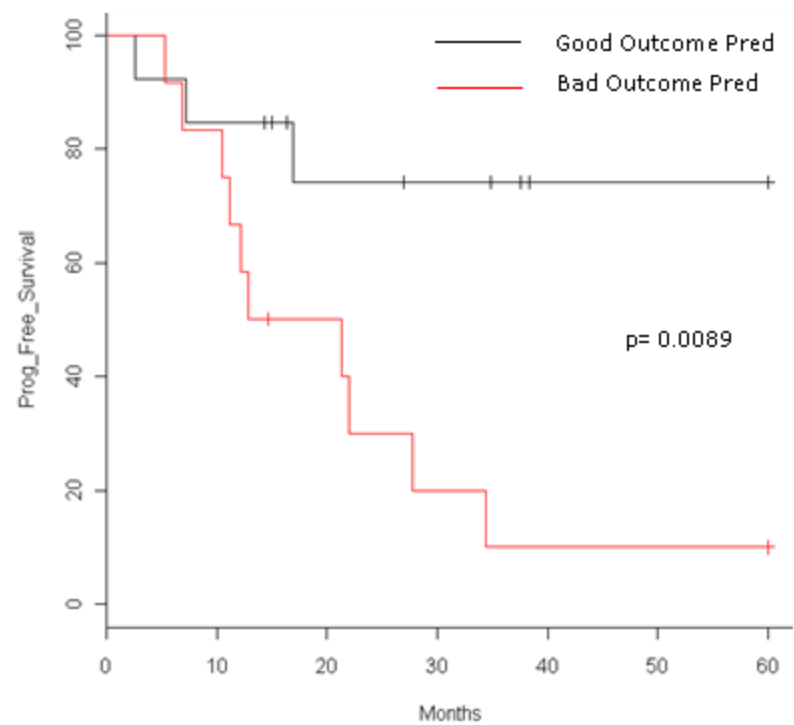

Supplement: Additional file 3 — Kaplan-Meier and log-rank analysis of patients with Stage 4 tumors stratified according to the NB-MuSE classifier. Kaplan-Meier and log-rank analysis for INSS Stage 4 neuroblastoma patients belonging to the external validation dataset. 5-years overall survival (left) and event free survival (right) of patients stratified according to the NB-MuSE classifier. Red and black curves represent poor and good outcome patients respectively. The p-value of the log-rank test is shown. [file 1471-2105-13-S4-S13-S3.pdf]

## ST1,ST2,ST3,ST4s

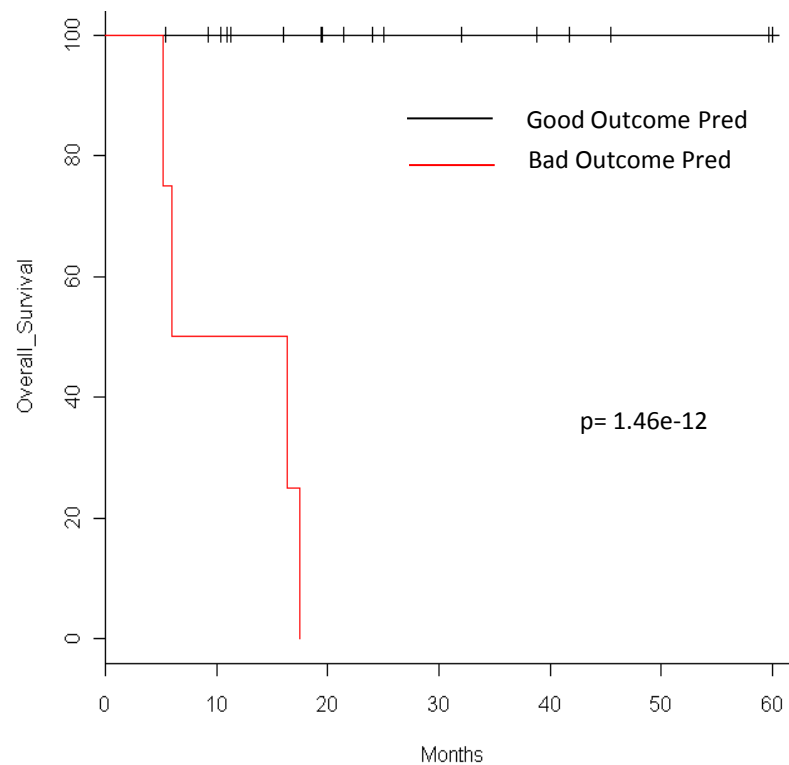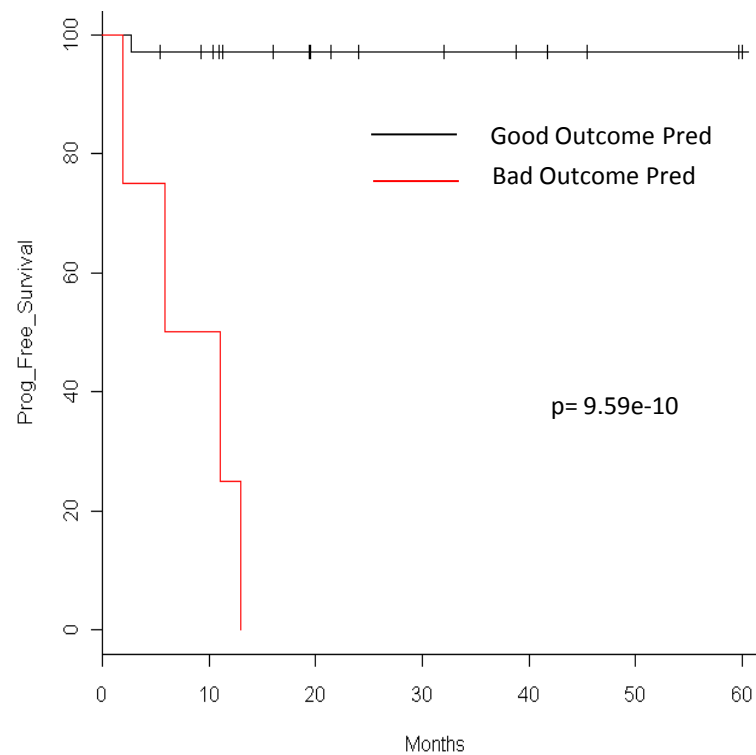

Supplement: Additional file 4 — Kaplan-Meier and log-rank analysis of patients with localized and Stage 4s tumors stratified according to the NB-MuSE classifier. Kaplan-Meier and log-rank analysis for INSS Stage 1,2,3 and 4s neuroblastoma patients belonging to the external validation dataset. 5-years overall survival (left) and event free survival (right) of patients stratified according to the NB-MuSE classifier. Red and black curves represent poor and good outcome patients respectively. The p-value of the log-rank test is shown. [file 1471-2105-13-S4-S13-S4.pdf]

# MYCN Amplified

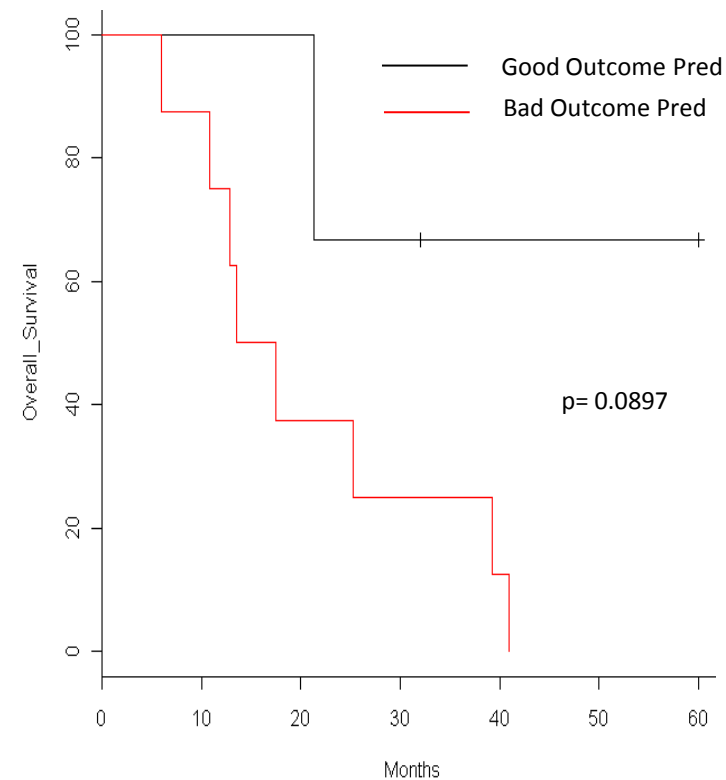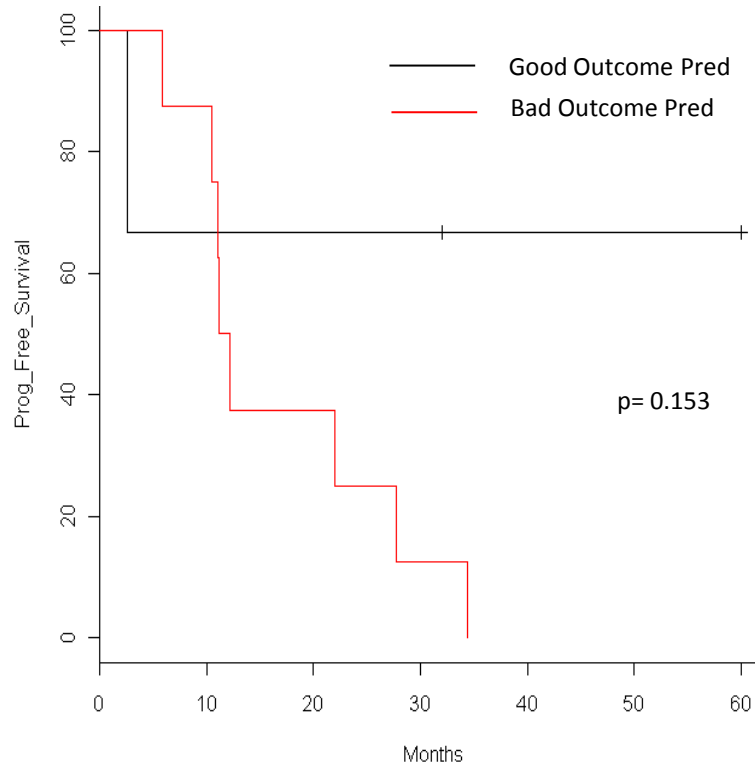

Supplement: Additional file 5 — Kaplan-Meier and log-rank analysis of patients with MYCN amplified tumors stratified according to the NB-MuSE classifier. Kaplan-Meier and log-rank analysis for neuroblastoma patients with MYCN amplified tumors belonging to the external validation dataset. 5-years overall survival (left) and event free survival (right) of patients stratified according to the NB-MuSE classifier. Red and black curves represent poor and good outcome patients respectively. The p-value of the log-rank test is shown. [file 1471-2105-13-S4-S13-S5.pdf]

## MYCN Not Amplified

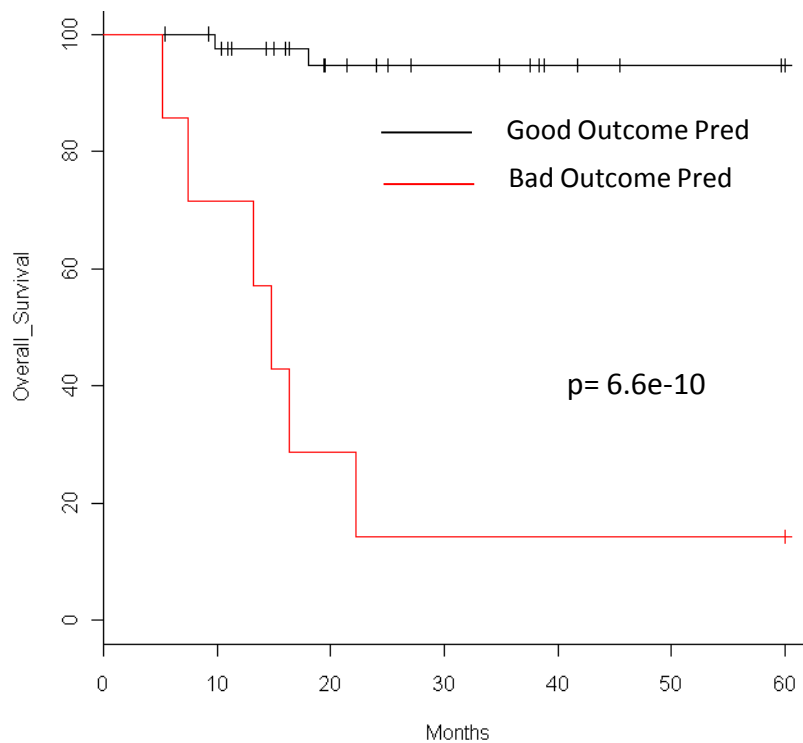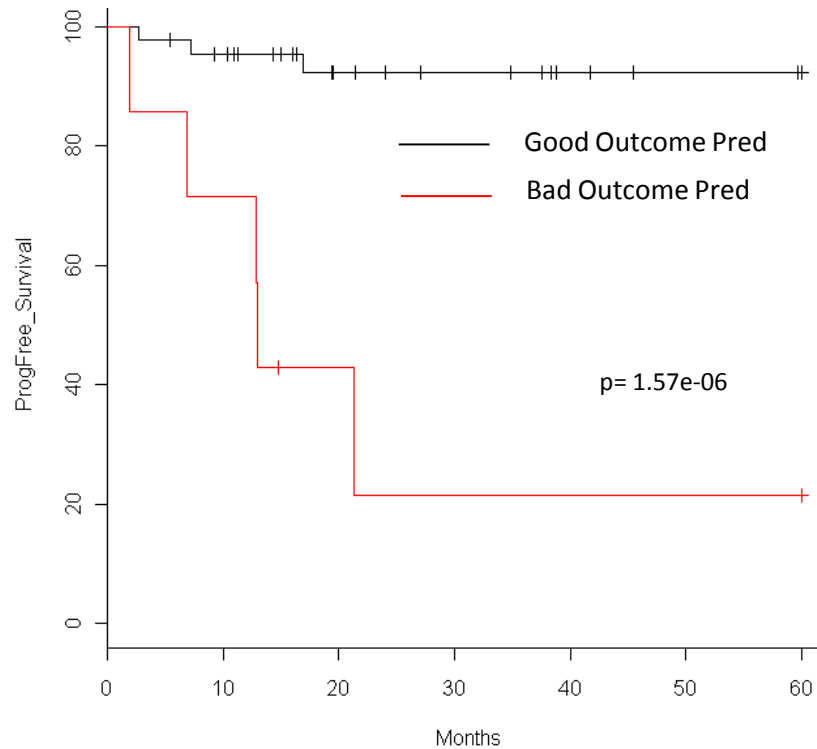

Supplement: Additional file 6 — Kaplan-Meier and log-rank analysis of patients without MYCN amplification in the tumors stratified according to the NB-MuSE classifier. Kaplan-Meier and log-rank analysis for neuroblastoma patients with MYCN not amplified tumors belonging to the external validation dataset. 5-years overall survival (left) and event free survival (right) of patients stratified according to the NB-MuSE classifier. Red and black curves represent poor and good outcome patients respectively. The p-value of the log-rank test is shown. [file 1471-2105-13-S4-S13-S6.pdf]
